# Patterns of host gene expression associated with harboring a foregut microbial community

**DOI:** 10.1186/s12864-017-4101-z

**Published:** 2017-09-06

**Authors:** Kevin D. Kohl, Kelly F. Oakeson, Diane Dunn, David K. Meyerholz, Colin Dale, Robert B. Weiss, M. Denise Dearing

**Affiliations:** 1 Department of Biological Sciences, Vanderbilt University, 465 21st Ave South, Nashville, TN 37235 USA; 2 Department of Biology, University of Utah, 257 South 1400 East, Salt Lake City, UT 84112 USA; 3 Department of Pathology, University of Iowa, 200 Hawkins Dr, Iowa City, IA 52242 USA; 4 Department of Human Genetics, University of Utah, 15 North 2030 East, Salt Lake City, 84112 USA

**Keywords:** Host-microbe interactions, Herbivore, Woodrat

## Abstract

**Background:**

Harboring foregut microbial communities is considered a key innovation that allows herbivorous mammals to colonize new ecological niches. However, the functions of these chambers have only been well studied at the molecular level in ruminants. Here, we investigate gene expression in the foregut chamber of herbivorous rodents and ask whether these gene expression patterns are consistent with results in ruminants. We compared gene expression in foregut tissues of two rodent species: Stephen’s woodrat (*Neotoma stephensi*), which harbors a dense foregut microbial community, and the lab rat (*Rattus norvegicus*), which lacks such a community.

**Results:**

We found that woodrats have higher abundances of transcripts associated with smooth muscle processes, specifically a higher expression of the smoothelin-like 1 gene, which may assist in contractile properties of this tissue to retain food material in the foregut chamber. The expression of genes associated with keratinization and cornification exhibited a complex pattern of differences between the two species, suggesting distinct molecular mechanisms. Lab rats exhibited higher abundances of transcripts associated with immune function, likely to inhibit microbial growth in the foregut of this species.

**Conclusions:**

Some of our results were consistent with previous findings in ruminants (high expression of facilitative glucose transporters, lower expression of *B4galnt2*), suggestive of possible convergent evolution, while other results were unclear, and perhaps represent novel host-microbe interactions in rodents. Overall, our results suggest that harboring a foregut microbiota is associated with changes to the functions and host-microbe interactions of the foregut tissues.

**Electronic supplementary material:**

The online version of this article (10.1186/s12864-017-4101-z) contains supplementary material, which is available to authorized users.

## Background

Complex and intimate associations with fermentative gut microbes have been instrumental in the success of mammalian herbivores [1, 2]. In general, these associations take place in enlarged gut compartments that house both the symbiotic microbes and the food material being digested. The most notable of these, the rumen, can compose 10–25% of an animal’s body weight, and the microbes housed within can provide 50–80% of the daily energetic needs of the host [2]. In addition to ruminants, a number of other animals exhibit foregut fermentation, such as some macropod marsupials, nonhuman primates, and rodents [2]. Understanding the evolution and function of foregut fermentation chambers is of considerable interest to biologists, because of its key role in facilitating herbivory.

The phenotypic and functional variation observed between species is a consequence of differences in gene expression as well as gene content [3]. Changes in gene expression profiles associated with hosting foregut microbial communities have been studied in a number of ways. First, developmental studies have compared gene expression in rumen epithelial tissue from suckling animals (before the establishment of fermentation) to adults [4–6]. Additionally, others have compared gene expression in tissues from various gut chambers, such as the fermentative rumen versus the acid-producing abomasum [7]. However, the question of which changes in gene expression are associated with hosting a foregut microbial community has not been evaluated in a comparative sense. Furthermore, this question has only been investigated in ruminants, and thus studies into the foregut chambers of other mammals may reveal potential convergence in gene expression profiles.

Here, we compare gene expression profiles in the proximal stomach regions of two rodent species that differ in diet, stomach morphology, and density of associated microbes. Woodrats (*Neotoma* spp.) are small herbivorous rodents that inhabit North America, and exhibit a bilocular, or semi-segmented stomach morphology [8]. We have demonstrated that the foregut harbors a dense microbial community (~10^10^ live cells/g contents) and has high concentrations of short chain fatty acids (~200 mM), which is a signature of microbial fermentation [9]. Conversely, laboratory rats are omnivorous, have a single-chambered stomach with fewer microbial cells (10^3−^10^4^; [10] and much lower concentrations of short chain fatty acids (<5 mM; [11]. The rat foregut region is thought to be largely for food storage, as surgical removal of this section does not impact food digestion [12]. The foreguts of both species are nonglandular and lined by keratinized, stratified squamous epithelia [8]. The current study focuses on a comparative analysis of foregut gene expression in order to gain insight into host-microbe interactions in the woodrat foregut.

Comparing the gene expression profiles from the foregut regions of woodrats and lab rats should reveal genes associated with hosting a foregut microbial community that participates in fermentation of food. We generated several hypotheses based on previous research in ruminant herbivores. First, given the evolution of bilocular morphology of the woodrat foregut, and the importance of muscular function in the mixing and passage of food material in ruminants [13, 14], we predicted altered expression of genes involved in smooth muscle function in the control of gastrointestinal motility. Second, the rumen of ruminants and foreguts of rats are both lined by keratinized, stratified squamous epithelium. In the rumen, tissues exhibit high levels of expression of genes in the mammalian epidermal development complex [7]; thus we predicted altered expression of genes associated with epidermal development and differentiation in woodrats compared to the lab rat foregut. Third, post-weaning development of the fermentative rumen coincides with increased expression of genes associated with fatty acid oxidation and ketogenesis [4–6]. Given the high concentration of short chain fatty acids in the woodrat foregut, we also predicted that this tissue would have higher expression of genes associated with fatty acid oxidation and ketogenesis. Fourth, post-weaning development of the rumen also produces significant changes in the expression of several solute carrier (*Slc*) genes, such as increases in the expression of transporters for glucose and urea [4, 6]. Therefore, we hypothesized that woodrats and lab rats would exhibit differential expression of *Slc* genes. Finally, we used untargeted techniques to investigate the data for differential abundances in other biological functions. Together, these approaches highlight convergent and divergent gene expression profiles associated with hosting a foregut microbial community.

## Methods

### Animals and tissue collection

Stephen’s woodrats (*Neotoma stephensi*) were collected near Wupatki National Monument, AZ, USA (35°30′ N, 111°27′ W) in October 2013. Woodrats were fed juniper foliage (*Juniperus monosperma*) during transport to the laboratory, for one evening in the laboratory, and were dissected the next day. Sprague Dawley lab rats were purchased from Harlan Laboratories (Madison, WI, USA), and individually housed in the open top cages in a room shared with woodrats (*Neotoma* spp., in separate open top cages). Lab rats were maintained in these conditions for ~6 months, being fed standard rat chow (Harlan Teklad formula 2018). While lab rats and woodrats were fed different diets in this experiment, the diets represent their natural foods. All animals used in our experiment were females (3 for each species). Lab rats used in this experiment were virgins; we do not know whether this was the case for wild-caught woodrats. All animals were euthanized with isoflurane. We collected a small section of the fornix ventricularis of the stomach (or the fundus of the stomach), which was immediately rinsed in ice-cold physiological saline buffer and placed in RNAlater solution. After 24 h, the RNAlater solution was removed and tissues were frozen at −80 °C.

### Library generation and RNA-Seq

Total RNA from each animal was isolated from frozen tissue samples using Qiagen RNeasy kit according to manufacturer instructions (Qiagen, Valencia, CA). An amount of 1.0 μg of total RNA from each animal was used to construct strand-specific, paired-end sequencing libraries from polyA-containing mRNA molecules using the Illumina TruSeq Stranded mRNA sample Preparation Kit (Illumina, Inc., San Diego, CA). Individual libraries were multiplexed and sequenced on a single lane of an Illumina HiSeq 2000 platform generating ~84 million total paired-end reads of 101 bp in length. Read quality and duplication levels were evaluated with the FastQC tool (https://www.bioinformatics.babraham.ac.uk/projects/fastqc). The average Phred value of trimmed Fastq sequence was 38, and the level of de-duplicated reads was in the expected range (34.4% to 42.3%) for a moderate level of rRNA and repeat reads from a total RNA library. Paired-end reads were quality filtered and trimmed using Trimmomatic [15] and the subsequent overlapping paired-end reads were merged using FLASH [16], which aligned ~90% of the read pairs into single, directional reads; the average insert size of the libraries was 139 bp, (SD = 3 bp.). These composite reads were merged with the forward reads from the remaining unmerged read pairs, resulting in ~46 million merged *N. stephensi* reads and ~37 million *R. norvegicus* reads. All reads have been uploaded to the NCBI Gene Expression Omnibus under accession GSE84381.

### A map and count pipeline using mouse CDS transcripts as the reference genome

Since de novo transcriptome assemblies are fragmented and incomplete, we chose first to align the composite reads from both *N. stephensi* and *R.norvegicus* to the protein-coding portion of the comprehensive gene annotation of *Mus musculus* from the GENCODE mouse C57BL6/J annotation project [17]. The coding transcripts from the M7 release of the GENCODE mouse genome annotation (gencode.vM7.pc_transcripts.fa) were formatted as a nucleotide database and Illumina reads were aligned using a fast version of the Smith-Waterman algorithm implemented in cross_match [18] with a -minscore parameter of 50 and a -masklevel parameter of 101 to allow reads to align to multiple transcripts. Total read counts were generated from read alignments to the correct strand per CDS region using custom Perl scripts that collapsed transcript level alignments (ENSMUST identifiers) to gene level alignments (ENSMUSG identifiers). Reads were assigned to a ENSMUSG gene identifier by their maximum cross_match alignment score, and reads that aligned to multiple genes with equivalent scores were evaluated for representation of paralogous genes, pseudogenes, overlapping genes and duplicate gene annotations. We excluded 519 ENSMUSG identifiers from the transcript database (Additional file 1: Table S1) based on cataloging multiple read alignments, and then re-evaluated the multiple read alignments to arrive at a final set of uniquely mapped read counts.

Multi-mapping reads for several key paralogous families encoding highly abundant transcripts relevant to epidermal differentiation were considered in a secondary analysis. In mammals, genes involved in the synthesis of the insoluble cornified envelope (CE) epithelial layer are found in a large gene cluster known as the epidermal differentiation complex or EDC; [7] that includes the Late Cornified Envelope Group I genes (*Lce1* gene symbols), the Late Cornified Envelope Group 3 genes (*Lce3* symbols), and the Cornifins, also known as small proline-rich region proteins (*Sprr1*, *Sprr2*, *Sprr3* and *Sprr4* families*)*. Keratins (all *Krt#* symbols) also occur in several large clusters of paralogous genes. Since cross-species mapping within these genes families is challenging due to gene duplications and homogenization within the EDC, we collapsed read counts to the family level for these genes. Differential gene expression analysis was performed on read counts using quasi-likelihood (QL F-test) methods in edgeR [19]. The tests for differential expression (DE) between experimental groups used the glmQLFTest function, and DE genes were identified at a false discovery rate (FDR) of 1%, using the Benjamini-Hochberg method for multiple testing correction.

### De novo transcriptome assembly

As no reference genome sequence exists for *Neotoma stephensi,* we used a novel de novo algorithm, *BinPacker* [20], to assemble a foregut reference transcriptome. The sequence input was 45,992,608 composite reads from the 3 *N. stephensi* foregut libraries, with default parameters for single-end, stranded reads, resulting in 44,311 putative transcripts with a median length of 332 bp, and a total length of 58.6 Mb. Assembled *N. stephensi* transcripts were used to validate the read counts generated by the Mouse CDS map and count pipeline.

### Testing a priori hypotheses

For smooth muscle genes, we used two transcriptome datasets from the mouse Smooth Muscle Cell Genome browser [21]. RNA-Seq Illumina reads were downloaded from the NCBI GEO database for mouse jejunum tissue (GSM1388412) and for sorted primary smooth muscle cells isolated from mouse jejunum tissue (GSM1388406).

To compare the representation of genes of various biological processes, we used the g:Cocoa tool within g:Profiler [22]. This tool compares gene lists and determines which biological functions are significantly enriched in those lists. The analysis can treat the gene lists as ordered, in which genes listed higher in the list are weighted more heavily [22]. The g:Cocoa tool also performs a custom multiple testing correction procedure, called g:SCS [23]. For our analysis we took the 1000 most differentially expressed genes from woodrats and lab rats, ordered from highest to lowest in terms of log_2_(fold change). We investigated all Biological Processes within Gene Ontology and used the default g:SCS multiple testing correction. For our a priori hypotheses, we investigated whether various Gene Ontology terms associated with (1) epidermal development and differentiation and (2) fatty acid oxidation and ketogenesis. Specific GO terms can be found in the Results section and Table 2.

Last, based on previous studies in ruminants, we were interested in differences in expression of solute carriers (*Slc*) genes. We determined the top five most differentially expressed *Slc* genes for each species, as determined by log(fold change), excluding mitochondrial transporters.

### Untargeted approach

We also wanted to compare expression using an untargeted approach to uncover other biological processes that may differ between the foreguts of woodrats and lab rats. First, we identified the top 10 differentially expressed genes in each species. We also used g:Cocoa to investigate other biological processes that are differentially enriched in the two gene lists (using the top 1000 most differentially expressed genes from each species as described above). We used the “moderate filtering” setting to select the most significantly enriched GO term within each parent term (since GO terms are hierarchical). We then sorted these by *P*-values, and present the 10 most highly enriched GO terms for both woodrats and lab rats.

### Histology

At the time of dissection, another section of the foregut stomach was prepared for histology by pinning the section to corkboard and then placing the pinned samples in glass vials containing 10% neutral-buffered formalin. Samples were shipped to the University of Iowa Comparative Pathology Laboratory for routine tissue processing, embedding, sectioning, and staining. Tissues were stained with 3,3′-diaminobenzidine (DAB) using a biotin-tagged *Dolichos biflorus* agglutinin (DBA) lectin. The DBA lectin binds to terminal, nonreducing N-acetylgalactosamine (GalNAc) residues produced by *B4galnt2* [24]. Additionally, we determined the relative muscle composition (muscularis mucosae, muscularis propria, and the sum of these two as an estimate of ‘total muscle’) of the foregut wall. For each tissue section, the area of each of these parameters was divided by the total area of the stomach wall to get a percentage of the total area.

## Results

**Fig. 1:**
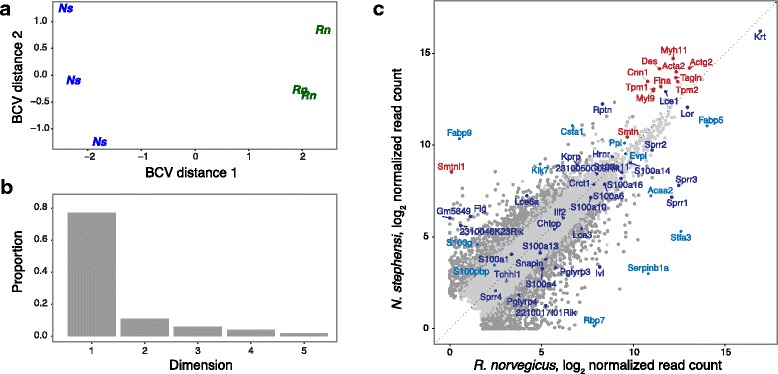
Relative foregut transcript abundances between the two rodent species. **a** MDS plot of the biological coefficient of variation (BCV) values over dimensions 1 and 2 with samples colored and labeled by sample groups. **b** Bar plot of the proportion of variance (eigenvalues) explained by the first 5 dimensions. **c** Normalized log_2_ read counts are shown for 11,870 detected genes, with transcripts that are statistically significant (FDR < 0.01) colored in dark grey. Specific classes of genes are highlighted, with smooth muscle-specific transcripts in red, epidermal differentiation complex transcripts in dark blue, and other genes involved in keratinocyte differentiation are shown in light blue

Read counts for protein-coding transcripts from the foregut tissues of *Neotoma stephensi* and *Rattus norvegicus* were obtained by RNA-Seq, aligned to the protein-coding portion of the comprehensive gene annotation of *Mus musculus,* and analyzed for differential abundance. Genes with expression below 5 counts-per-million (CPM) in three or more samples were removed and model-based normalization was performed with edgeR to eliminate composition bias between libraries, resulting in 11,870 detected transcripts. A multi-dimensional scaling analysis indicated that the *N. stephensi* and *R. norvegicus* grouping best separated the top 1500 genes that have the largest variation between the RNA-Seq libraries (Fig. 1a), and 77% of the variance was explained by this grouping (Fig. 1b). The biological coefficient of variation (CV) of 34% (edgeR biological CV) was consistent with CV estimates seen between biological replicates with outbred samples [25]. The Spearman rank correlation between the lab rat and woodrat transcriptomes was 0.78. A total of 2306 genes showed significant differential abundance between *N. stephensi* and *R. norvegicus* at a false discovery rate (FDR) < 0.01: 1157 genes were more abundant and 1149 genes were less abundant in *N. stephensi* versus *R. norvegicus* foregut tissue (Fig. 1c; Additional file 1: Table S2).

### Validation of analysis pipeline

It was important to judge the average nucleotide sequence divergence contained in woodrat:mouse versus the lab rat:mouse alignments. The mean nucleotide substitution per read from the *cross_match* alignments was 7.0% (*s* = 2.2%) for the *N. stephensi* alignments and 5.4% (*s* = 1.9%) for *R. norvegicus*, with similar distributions observed for mRNAs within the differentially abundant classes (Additional file 2: Figure S1), indicating that the cross-species mapping pipeline did not have global, systematic bias in mapping *N. stephensi* and *R. norvegicus* reads to mouse transcripts and annotations.

**Fig. 2:**
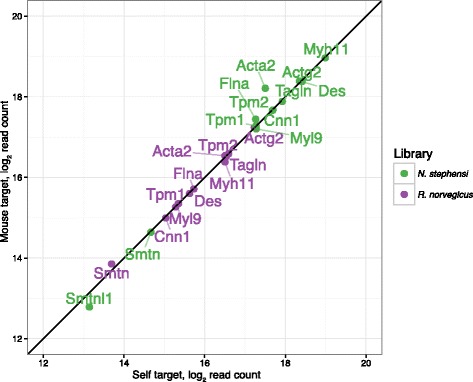
Smooth muscle-specific genes display only minor deviations when mapped within-species versus cross-species. The x-axis shows the read count from cross_match alignments to ‘self’ targets comprising within-species CDS region transcripts from *R. norvegicus* Ensembl gene predictions or *N. stephensi* de novo assemblies from BinPacker or Trinity. The y-axis shows the read count from alignments to the *M. musculus* GENCODE transcripts. The *R. norvegicus Smtnl1* (smoothelin-like 1 transcript) is not shown because only one read was detected in the cross-species mapping and zero reads were detected in the within-species mapping. By comparison, for the *N. stephensi Smtnl1* transcripts, 7064 reads were detected in the cross-species alignments and 9050 reads in the within species alignments

Our methods were also validated using read alignments to ‘self’ CDS sequences from both species chosen from Ensembl gene annotation (laboratory rat) or from de novo assembly of the *N. stephensi* foregut RNA-Seq reads. The Pearson correlation coefficient for read counts between the ‘self’ mapping versus the mouse CDS ‘map and count’ pipeline was 0.98 (Fig. 2), indicating that the *cross_match* ‘map and count’ pipeline accurately quantitated abundance from the cross-species CDS alignments to the orthologous mouse annotation. Last, the global validity of the cross-species ‘map and count’ pipeline was also examined for read mapping bias that may have confounded the observed abundance differences. We mapped short reads obtained from mouse jejunum smooth muscle to both the GENCODE vM7 CDS transcript database using the ‘map and count’ pipeline and to mouse genomic reference sequence (mm10) using the STAR aligner [26]. After extracting coding sequence read counts from the genomic STAR alignment and comparing these with read counts from the ‘map and count’ pipeline, the Spearman’s rank correlation was 0.98 for 11,192 detected genes in mouse jejunum. The minor differences in the approach resulted from how each pipeline adjudicates multi-mapping reads and indicated that our transcript mapping pipeline was robust for RNA-Seq data from a relevant tissue.

### A priori hypotheses

**Fig. 3:**
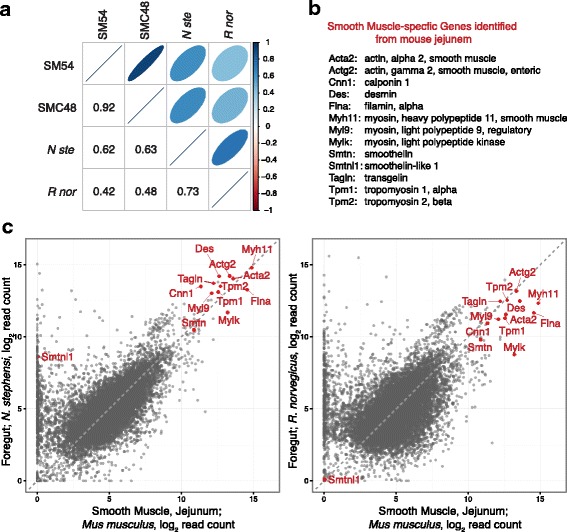
RNA-Seq transcriptomes compared between mouse smooth muscle tissue/cells versus woodrat and laboratory rat foregut tissues. The mouse smooth muscle (SM) RNA-Seq data was obtained from NCBI: GSM1388412, (SM Jejunum) and GSM1388406 (SM cells Jejunum). **a** A Spearman rank correlation matrix of read counts from 10,241 genes detected in woodrat (N ste) or lab rat (R nor) foregut compared with mouse smooth muscle tissue (SM54 = GSM1388412) or isolated mouse smooth muscle cells (SMC48 = GSM1388406). **b** List of smooth muscle-specific transcripts identified from the Smooth Muscle Cell Genome Browser [21]. **c** Normalized log_2_ read count scatter plots showing the comparative transcript abundance between mouse SM jejunum and the rat foregut tissues, with smooth muscle-specific transcripts shown in red

Scatterplots of individual transcript abundance indicated that smooth muscle-specific genes were more abundant as a class in *N. stephensi* foregut, especially smoothelin like-1 (*Smtnl1*; Fig. 1c)*.* We also compared transcript abundances to the transcriptome of sorted primary smooth muscle cells isolated from mouse jejunum tissue. The Spearman rank correlation between read counts from mouse jejunum smooth muscle tissue compared to the woodrat foregut tissue was 0.62 (Fig. 3a), versus 0.42 for the laboratory rat foregut, supporting the increased smooth muscle composition of the woodrat foregut. We identified smooth muscle-specific transcripts (Fig. 3b), and compared abundances of these transcripts between the mouse smooth muscle transcriptome and our rodent foregut samples (Fig. 3c). Woodrats exhibited a general increase in transcripts encoding the abundant proteins of the contractile apparatus and a unique increase in the smoothelin-like-1 (*Smtnl1*) transcript that encodes a protein involved in regulating the contractile properties of smooth muscle [27].

Epidermal-specific genes showed a more complex pattern of specific changes (Fig. 1c)*.* There was no significant enrichment for the following Gene Ontology terms: (GO:0009913: epidermal cell differentiation; GO:0008544: epidermis development) when using g:Cocoa to investigate enrichment in functional categories. However, the term “keratinocyte differentiation” (GO:0030216) was significantly enriched in the woodrat foregut compared to the lab rat foregut (*P* = 0.03). To focus on differentially expressed epidermal genes, we used the mouse jejunum smooth muscle RNA-Seq data as a filter to identify 510 transcripts not found in smooth muscle (Additional file 1: Table S3 and S4, Additional file 2: Figure S2). We observed 124 epidermal-specific transcripts that were differentially abundant. Specifically, we observed enrichment of cornefin genes (*Sprr1* and *Sprr3* family) abundance (Fig. 1c, Additional file 1: Table S5) in laboratory rat foregut, while woodrats exhibited higher expression of filaggrin and repetin (Fig. 1c, Additional file 1: Table S5). There was also highly abundant expression of fatty acid-binding protein (*Fabp9*) in the *N. stephensi* foregut, a gene that is restricted in expression to male germ-cells in lab mice [28]. Woodrats exhibited high-level expression of the known epidermal fatty acid-binding protein (*Fabp5*). Phylogenetic analysis of the *N. stephensi Fabp5* and *Fabp9* genes confirmed the identity of the transcripts quantitated by RNA-Seq (Additional file 2: Figure S3).

We found little evidence supporting the prediction that woodrats would exhibit higher expression of genes associated with fatty acid oxidation and ketogenesis. There were no significant differences between woodrats and lab rats in representation of several Gene Ontology terms related to ketogenesis and response to ketones (GO:1,901,654: response to ketone; GO:0042180: cellular ketone metabolic process; GO:0042181: ketone biosynthetic process; GO:1,901,655: cellular response to ketone). Similarly, we found no differences in several GO terms related to the metabolism of fatty acids (GO:0019395: fatty acid oxidation; GO:0006631: fatty acid metabolic process; GO:1,901,568: fatty acid derivative metabolic process; GO:0033559: unsaturated fatty acid metabolic process). However, woodrats were significantly enriched in genes associated with long-chain fatty acid metabolic process (GO:0001676; *P* = 0.045), and exhibited higher expression of fatty acid-binding protein 9 (*Fabp9;* Fig. 1c).

A number of solute carrier proteins exhibited significant differential expression between woodrats and lab rats. We compared these differences to what has been previously observed in young, non-fermenting ruminants compared to older, fermenting ruminants, but were unable to find any consistent similarities (Table 1). More specifically, in both species there were similar numbers of *Slc* genes that were consistent with the results observed in ruminants (3/4 in woodrats and 2/4 in lab rats). Urea transporters (*Slc14*), which are important for nitrogen recycling, showed no differential expression between the two species.

**Table 1:** The five most differentially expressed solute carrier genes in woodrats and lab rats

Gene symbol	Description	Log_2_(Fold Change)	Consistent with prediction from ruminants?
Woodrats			
*Slc41a2*	Magnesium transporter	7.69	–
*Slc16a10*	Monocarboxylic acid transporter	5.59	Yes
*Slc12a2*	Sodium/potassium/chloride transporter	3.67	No
*Sla2a10*	Facilitated glucose transporter	2.74	Yes
*Slc2a12*	Facilitated glucose transporter	2.49	Yes
Lab Rats			
*Slc6a20a*	Proline IMINO transporter	7.63	Yes
*Slc19a3*	Thiamine transporter	5.78	No
*Slc27a6*	Fatty acid transporter	5.74	No
*Slc23a3*	Nucleobase transporter	5.20	–
*Slc1a1*	Glutamate transporter	5.19	Yes

Predictions for ruminants were based on previous research [4], and we sometimes compared SLC genes in the same family (for example, *Slc16a10* in woodrats and *SLC16A1* in cattle, which are both monocarboxylic acid transporters)

### Untargeted approach

We also pushed beyond our a priori approach by testing for differentially expressed genes across the transcriptome. First, we present the top 10 differentially expressed genes in each animal (Table 2). A number of these genes support our hypotheses, such as woodrats exhibiting higher expression of smoothelin-like 1 (*Smtnl1*; a gene associated with muscle tissues) and fatty acid binding protein 9 (*Fabp9*). Lab rats exhibited higher expression of the phospholipase A2, Group IIA (Pla2g2a) and beta-1,4-N-acetyl-galactosaminyl transferase 2 (*B4galnt2*) genes.

**Table 2:** The top ten differentially expressed genes from the foregut tissues of each species, as determined by log_2_(fold change)

Gene symbol	Gene name	Log_2_ (Fold Change)	FDR-corrected P-value
Overexpressed in woodrats			
*Smtnl1*	Smoothelin-like 1	12.34	1.46E-04
*Krt24*	Keratin 24	11.91	4.04E-03
*Fabp9*	Fatty Acid Binding Protein 9	11.54	1.02E-03
*Mpp1*	Membrane Protein, Palmitoylated 1	11.41	3.26E-04
*Pnck*	Pregnancy Up-Regulated Nonubiquitous CaM Kinase	10.89	1.74E-03
*Calcoco2*	Calcium Binding And Coiled-Coil Domain 2	10.47	3.23E-04
*Krt19*	Keratin 19	9.96	4.45E-04
*Ube2d4*	Ubiquitin-Conjugating Enzyme E2D 4	9.52	1.57E-04
*Mansc4*	MANSC Domain Containing 4	9.36	5.45E-04
*Akr1b7*	Aldo-keto reductase family 1, member B7	9.30	2.09E-04
Overexpressed in lab rats			
*Pla2g2a*	Phospholipase A2, Group IIA	12.23	1.46E-04
*Krt12*	Keratin 12	11.18	6.56E-05
*Rbp7*	Retinol Binding Protein 7	10.95	1.31E-04
*B4galnt2*	Beta-1,4-N-Acetyl-Galactosaminyl Transferase 2	10.40	1.01E-03
*Rnase1*	Ribonuclease, RNase A Family, 1	10.21	1.07E-03
*Aoc1*	Amine Oxidase, Copper Containing, 1	10.12	3.68E-04
*Gtpbp6*	GTP Binding Protein 6	10.10	9.78E-04
*Mybpc1*	Myosin Binding Protein C	10.03	9.47E-05
*Casp4*	Caspase 4	10.00	4.12E-04
*Fbp2*	Fructose-1,6-Bisphosphatase 2	10.00	5.45E-04

We also used g:Cocoa to determine the most over-represented GO terms in each animal (Table 3). In woodrats, genes related to development and muscle function were overrepresented (i.e. Actin cytoskeleton organization, Muscle contraction). In lab rats, several GO categories related to immune function were highly overrepresented. (i.e. Immune response, Positive regulation of T cell activation, Neutrophil migration).

**Table 3:** The top ten most overrepresented Biological Functions in each species, determined using g:Cocoa

Function	GO Term	No. genes in term	No. genes overexpressed in woodrats	No. genes overexpressed in lab rats
Overrepresented in woodrats				
Cellular component organization	GO:0016043	5139	347	252
Metabolic process	GO:0008152	11,355	638	580
Actin cytoskeleton organization	GO:0030036	520	65	18
Intracellular signal transduction	GO:0035556	2241	176	1
Cellular component morphogenesis	GO:0032989	1204	112	29
Regulation of multicellular organismal process	GO:0051239	2423	175	62
Regulation of molecular function	GO:0065009	2460	174	61
Regulation of intracellular signal transduction	GO:1,902,531	1417	107	1
Cell migration	GO:0016477	1081	53	55
Muscle contraction	GO:0006936	240	25	3
Overrepresented in lab rats				
Single-organism metabolic process	GO:0044710	4526	265	305
Immune response	GO:0006955	1107	10	69
Defense response	GO:0006952	1264	20	65
Organic hydroxy compound metabolic process	GO:1,901,615	431	22	43
Positive regulation of cell activation	GO:0050867	250	8	21
Positive regulation of T cell activation	GO:0050870	155	5	16
Neutrophil migration	GO:1,990,266	94	1	11
Nuclear DNA replication	GO:0033260	15	1	6
L-serine biosynthetic process	GO:0006564	4	0	4
Localization	GO:0051179	5203	289	305

Corrected *p*-values are all less than 0.001. We used the moderate filtering to first identify the most differentially represented term per parent group

### Histology

**Fig. 4:**
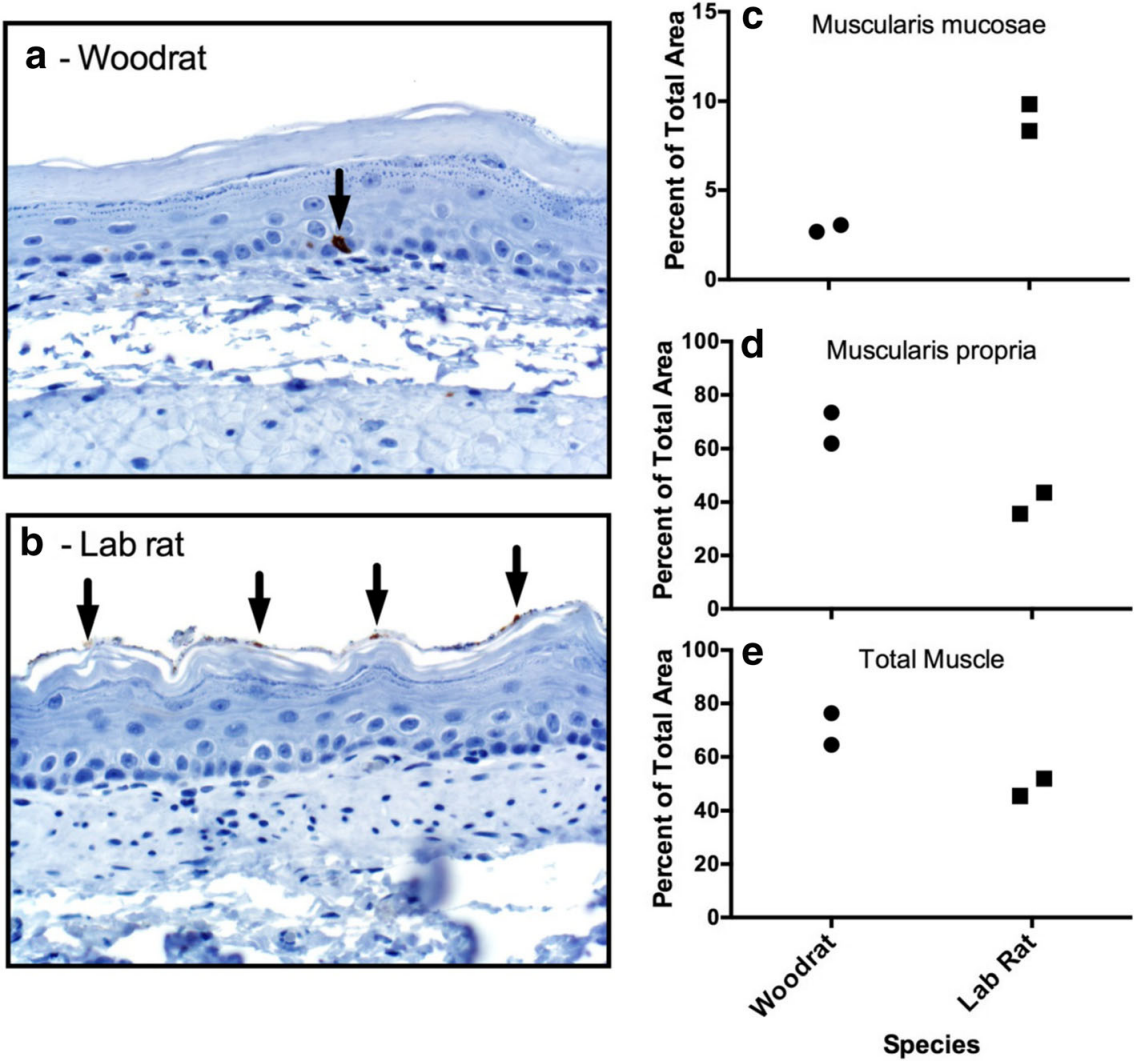
Histological and morphometric evaluation of tissues. **a**, **b** DBA lectin staining for the presence of activity of *B4galnt2*. Arrows point to cellular staining. **c**-**e** Percent of the total tissue wall that was composed of muscularis mucosae, muscularis propria, and total muscle (the sum of the two)

We were interested in whether we could find histological support for some results uncovered through RNA-Seq analysis. For example, the gene *B4galnt2* was more highly expressed in lab rat tissues (Table 2). In corroboration of this result, DBA lectin binding, which is specific for *B4galnt2*-carbohydrates, detected only scattered uncommon basal cells in the foregut of woodrats (Fig. 4a), whereas DBA lectin binding in lab rats was commonly detected in the cornified epithelial layer of the foregut (Fig. 4b).

Additionally, we found that genes associated with muscle activity were more highly expressed in the woodrat tissue (*Smtnl1:* Table 2, Table 3). We measured the proportion of the tissue wall in each species that was composed of various muscle tissues. Though our sample size was limited, we found that lab rats have a greater proportion of the muscularis mucosae (Fig. 4c), but woodrats have a greater proportion of muscularis propria and total muscle (Fig. 4d and e).

## Discussion

We compared gene expression in the foregut tissues of woodrats and lab rats, which differ in diet, stomach morphology, and density of associated microbes. The expression profiles of these two species were substantially different, highlighting the differential organization and function of the foregut tissue. We are unable to disentangle the many effects that could be underlying these differences in gene expression. They may be driven by diet, given that woodrats are herbivorous and lab rats are omnivorous. Resistant starches and dietary fiber can influence intestinal gene expression, though these differences may be modulated through the microbiota [29, 30]. Unfortunately, controlling for diet was not possible in this study, given that placing herbivorous rodents (including woodrats) on omnivorous diets can induce diabetes and other metabolic syndromes [31, 32]. Differential gut morphology also likely underlie the observed differences, given that woodrats have thicker muscle layer, and thus we observed higher abundance of genes associated with musculature (discussed below). Last, microbial communities are known to induce changes in host gene expression [33]. However, germ-free woodrats have not been developed, so we cannot control for these effects. It should also be noted that microbial communities [34] and host physiology [35] can change as an effect of captivity. Regardless of the exact mechanisms, the differences in gene expression between these two species reveal differential biological functions. We discuss our findings in relation to previous work conducted in ruminant systems.

Genes related to muscle system processes were over-represented in woodrat foregut tissue. This is likely due to the fact that a larger proportion of the woodrat foregut wall is composed of muscle tissue when compared to lab rats. In ruminants, muscle tissue is important for the mixing and passage of food material [13, 14]. Woodrats do not have a physical separation between stomach regions, as is observed in ruminants [8]. Thus, it could be that peristaltic movements in the woodrat foregut are important for the mixing and retention of food material in the foregut for microbial digestion. However, it should be noted that retention of food material in this chamber is relatively short (typically less than 1.5 h; [9]). Of particular interest is the increased level of smoothelin like-1, which regulates smooth muscle contraction and relaxation, and changes in expression levels are known to alter vascular response to exercise, as well as uterine smooth muscle activity during pregnancy [36]. The up-regulation of *Smtnl1* in woodrat foregut may therefore be a specific adaptation facilitating increased foregut motility.

In ruminants, the epithelial lining is composed of keratinized and stratified squamous epithelium, which has histological similarities to mammalian skin [37]. Rumen tissue exhibits high expression of a number of genes associated with epidermal development, especially a homolog of small proline-rich proteins (*Sprr*) and a trichohyalin-like gene [7]. Interestingly, the Sprr genes are highly expressed in sheep rumen, but not skin tissue [7]. In the current study, we found that the woodrats and lab rats had differential abundance of a number of genes associated with epidermal differentiation and development. From the EDC gene cluster, the *Sprr1* and *Sprr3* genes and the involucrin gene showed higher expression in the lab rat foregut. Since the Sprr proteins play known roles in modulating the barrier function and biomechanical properties of the epithelial surface, these abundance differences may also contribute to functional differences in barrier response to gut microflora. The woodrat foregut exhibited higher expression of repetin (*Rptn*), filaggrin (*Flg*), and cystatin A1 (*Csta1*) genes associated with the cornified envelope [38–40]. Together, these results suggest the molecular basis of this cornification may differ between lab rats and woodrats.

In ruminants, the rumen tissue itself utilizes short chain fatty acids (SCFAs) as energy substrates [41], and thus exhibits high expression of genes associated with metabolizing SCFAs [4–6]. We found no support for the hypothesis that woodrat tissues would have higher expression of genes related to ketogenesis and metabolism of fatty acids, even though this chamber contains high concentrations of SCFAs (~200 mM; [9]) relative to lab rats. Though, concentrations in the gut lumen are the result of both fatty acid production by microbes and absorption by the host. Rumen tissue exhibits higher rates of fatty acid absorption compared to the equivalent stomach regions in pigs and horses [2], and in horses, the gastric muscosa exhibits higher rates of SCFA absorption than the foregut region [42]. Thus, one potential explanation could be that there is low capacity for SCFA absorption in the woodrat foregut, and thus SCFAs may be absorbed and utilized by subsequent gut regions. Alternatively, SCFAs could be absorbed in the woodrat foregut, and be passed into the blood stream and metabolized in the liver, as occurs in other non-ruminant species [43]. This process might be facilitated by the high expression of fatty acid binding proteins, which are largely considered to be lipid chaperones [44]. In addition to high-level expression of *Fabp5* in both rodent species (a known epidermal fatty acid binding protein), woodrats specifically expressed high levels of *Fabp9*, whose tissue specific expression in other mammals is restricted to testis, where it is the most abundant protein of the sperm perinuclear theca [28]. Physiological studies investigating the absorption and utilization rates of SCFAs by woodrat foregut tissues would disentangle these possibilities.

We observed a number of differences in the types of transporters expressed in the foregut tissues of woodrats and lab rats. Woodrats exhibited higher expression of a monocarboxylic acid transporter (*Slc16a10*). Proteins from the *Slc16a* family are thought to be important for the absorption of SCFAs in rumen tissue [45], and thus similar absorption may be occurring in the woodrat foregut. Additionally, woodrats exhibited higher expression of two facilitative glucose transporters (*Slc2a10, Slc2a12*), which transport glucose across the basolateral membrane into the blood stream [46]. The expression of facilitative glucose transporters increases over development of ruminants, concordant with the development of fermentation [4]. High expression of these transporters in ruminants (and woodrats) is puzzling, given that apical absorption of glucose is presumed to be low in ruminants, since simple carbohydrates would largely undergo fermentation in the rumen [2]. Therefore, the role of facilitative transporters in the foregut epithelia of ruminants and woodrats remains unclear.

The foregut region of lab rats was highly enriched in genes associated with immune responses. The rats used in our study were healthy and not immune-challenged, and thus we assume this difference represents a higher basal expression of immune-related genes in this tissue. Tolerance, or ‘unresponsiveness’ to microbes is presumed to be important for allowing gut microbes to reside within the gastrointestinal tract [47]. Therefore, woodrats may have a decreased immune response to microbes in the foregut, allowing the resident microbial community to flourish. Consistent with this notion is the higher expression of ribonuclease (*Rnase1*) in the lab rat foregut, which is considered to have antimicrobial activity in the gut [48]. The *Pla2g2a* gene encoding the Group IIA secretory phospholipase A2 (sPLA2 IIA) is a well-known biomarker for inflammatory disease and was highly expressed in the lab rat while undetectable in the woodrat tissues. sPLA2 enzymes convert phospholipids used in the formation of extracellular lamellar membranes and participate in the acidification of the stratum corneum, important for antimicrobial defense [49], and mammalian sPLA2 IIA also specifically displays potent enzymatic antibacterial activity [50]. Together, these immune-related and antimicrobial genes may be important in suppressing microbial growth in the foregut chamber of lab rats. Gene knock-out studies in lab mice could be conducted to better investigate the connections between immune-related genes and microbial abundance in the foregut chamber.

Another interesting gene expressed at higher levels in the lab rat foregut tissue compared to woodrats was beta-1,4-N-acetyl-galactosaminyl transferase 2, or *B4galnt2.* This gene plays a role in the formation of host glycans, and is known to have long-term balancing selection of expression variation in rodents [51, 52]. Host glycans are thought to be important for the regulation of microbial communities [53], and indeed, knockout mice (*B4galnt2* ^*−/−*^) harbor distinct gut microbial communities compared to wild-type mice [51]. Differential *B4galnt2* expression in lab rats was supported by DBA lectin staining, which also revealed differential localization, such that lab rats maintain GalNAc residues on the epithelial surface, while woodrats do not. Interestingly, there is variation in the localization of *B4galnt2* expression and GalNAc residue localization across wild populations of mice, which is thought to be due to balancing selection between susceptibility to gut pathogens and a bleeding disorder [51, 52]. Additionally, in ruminants expression of *B4galnt2* significantly decreases with the development of a fermenting rumen [4]. The function and consequences of reduced *B4galnt2* expression in the woodrat foregut is an interesting area of future research.

## Conclusions

Together, these results inform us about the underlying changes in gene expression that are associated with hosting a foregut microbial community. We observed a number of differences that are consistent with previous findings in ruminants (high expression of facilitative glucose transporters, lower expression of *B4galnt2*). Additionally, a number of differences were observed between ruminants and rodents. For example, the molecular basis of cornification requires further study, and it is still unclear whether the capacity of woodrat foregut tissue to absorb and oxidize SCFAs is similar to that of the rumen. It would be interesting to conduct studies similar to this in other rodents with more developed foregut chambers. For example, a number of rodent genera actually have macrovilli in their stomachs that support microbial attachment of symbiotic microbes; [54], as well as in foregut fermenting primates and macropod marsupials. Together, these studies would reveal the shared changes in gene expression associated with hosting a foregut microbial community and shed light on the evolution of these microbial symbioses.

## Additional files

Additional file 1: Supplementary Tables. **Table S1**: Excluded GENCODE vM7 mouse genes. **Table S2**: EdgeR differential expression (*N. stephensi* versus *R. norvegicus*). **Table S3**: Read counts from GENCODE vM7 map and count pipeline: *M. musculus* smooth muscle versus *N. stephensi* and *R. norvegicus* foregut. **Table S4**: EdgeR differential expression (*N. stephensi* versus *R. norvegicus*); non-smooth muscle genes, FDR < 0.002. **Table S5**: EdgeR differential expression (*N. stephensi* versus *R. norvegicus*); keratins (Krt) and EDC (Sprr and Lce) genes collapsed to family gene symbols. (XLSX 3226 kb)

Additional file 2: Supplementary Figures. **Figure S1**. Average nucleotide divergence in mapped reads by differential abundance class for detected transcripts. The density plot summarizes the average substitution rate per read in the cross_match alignments of *N. stephensi* and *R. norvegicus* reads mapped to mouse 10,242 proteincoding sequences. The substitution per gene (SDI) was calculated as the sum of the cross_match substitution, insertion and deletion frequency per aligned read. **Figure S2**. Differential abundance of 510 epidermal-specific transcripts between the two rodent species. Genes that had at least 100X higher average expression levels in rat foregut versus mouse jejunem are shown, with labels and color (blue) indicating differentially abundant (FDR < 0.002) genes. **Figure S3**. Multiple sequence alignment and phylogenetic tree of the abundant fatty acid binding proteins (Fabp5 and Fabp9) found in woodrat and laboratory rat foregut tissue. Protein sequences from Fabp5 and Fabp9 from *N. stephensi*, *R. norvegicus* and *M. musculus* were aligned with MAFFT Multiple Sequence Alignment Software Version 7 using the L-INS-I algorithm (mafft --reorder –auto input). A) Jalview alignment is shown with amino acid restudies colored according to their physicochemical properties and B) phylogenetic tree based on Neighbor Joining algorithm with a distance matix determined from BLOSUM 62 score. (PDF 1407 kb)
