# Supplementary material for: Patterns of host gene expression associated with harboring a foregut microbial community
Source: BMC Genomics. 2017 Sep 6;18:697. doi: 10.1186/s12864-017-4101-z (PMC5585965; doi:10.1186/s12864-017-4101-z)
Supplement: Supplementary file 2 — Figure S1. Average nucleotide divergence in mapped reads by differential abundance class for detected transcripts. The density plot summarizes the average substitution rate per read in the cross_match alignments of N. stephensi and R. norvegicus reads mapped to mouse 10,242 proteincoding sequences. The substitution per gene (SDI) was calculated as the sum of the cross_match substitution, insertion and deletion frequency per aligned read. Figure S2. Differential abundance of 510 epidermal-specific transcripts between the two rodent species. Genes that had at least 100X higher average expression levels in rat foregut versus mouse jejunem are shown, with labels and color (blue) indicating differentially abundant (FDR < 0.002) genes. Figure S3. Multiple sequence alignment and phylogenetic tree of the abundant fatty acid binding proteins (Fabp5 and Fabp9) found in woodrat and laboratory rat foregut tissue. Protein sequences from Fabp5 and Fabp9 from N. stephensi, R. norvegicus and M. musculus were aligned with MAFFT Multiple Sequence Alignment Software Version 7 using the L-INS-I algorithm (mafft --reorder –auto input). A) Jalview alignment is shown with amino acid restudies colored according to their physicochemical properties and B) phylogenetic tree based on Neighbor Joining algorithm with a distance matix determined from BLOSUM 62 score. (PDF 1407 kb) [file 12864_2017_4101_MOESM2_ESM.pdf]

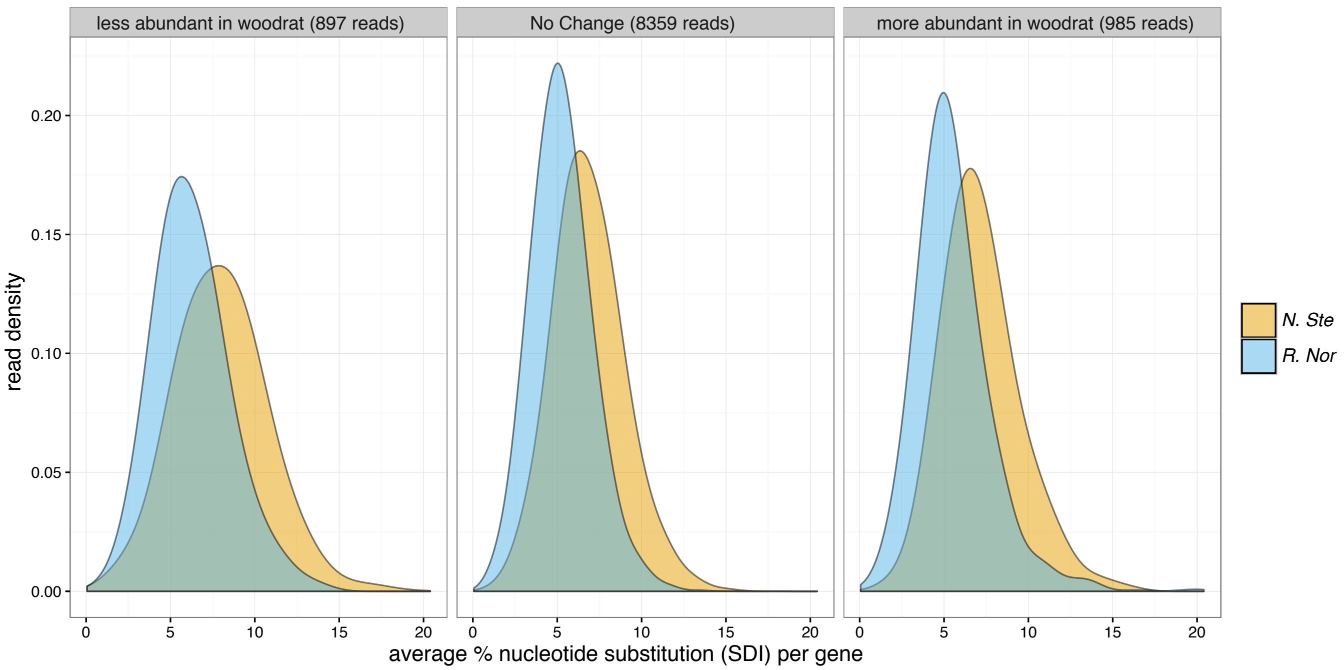

Figure S1. Average nucleotide divergence in mapped reads by differential abundance class for detected transcripts. The density plot summarizes the average substitution rate per read in the cross_match alignments of N. stephensi and R. norvegicus reads mapped to mouse 10,242 proteincoding sequences. The substitution per gene (SDI) was calculated as the sum of the cross_match substitution, insertion and deletion frequency per aligned read.

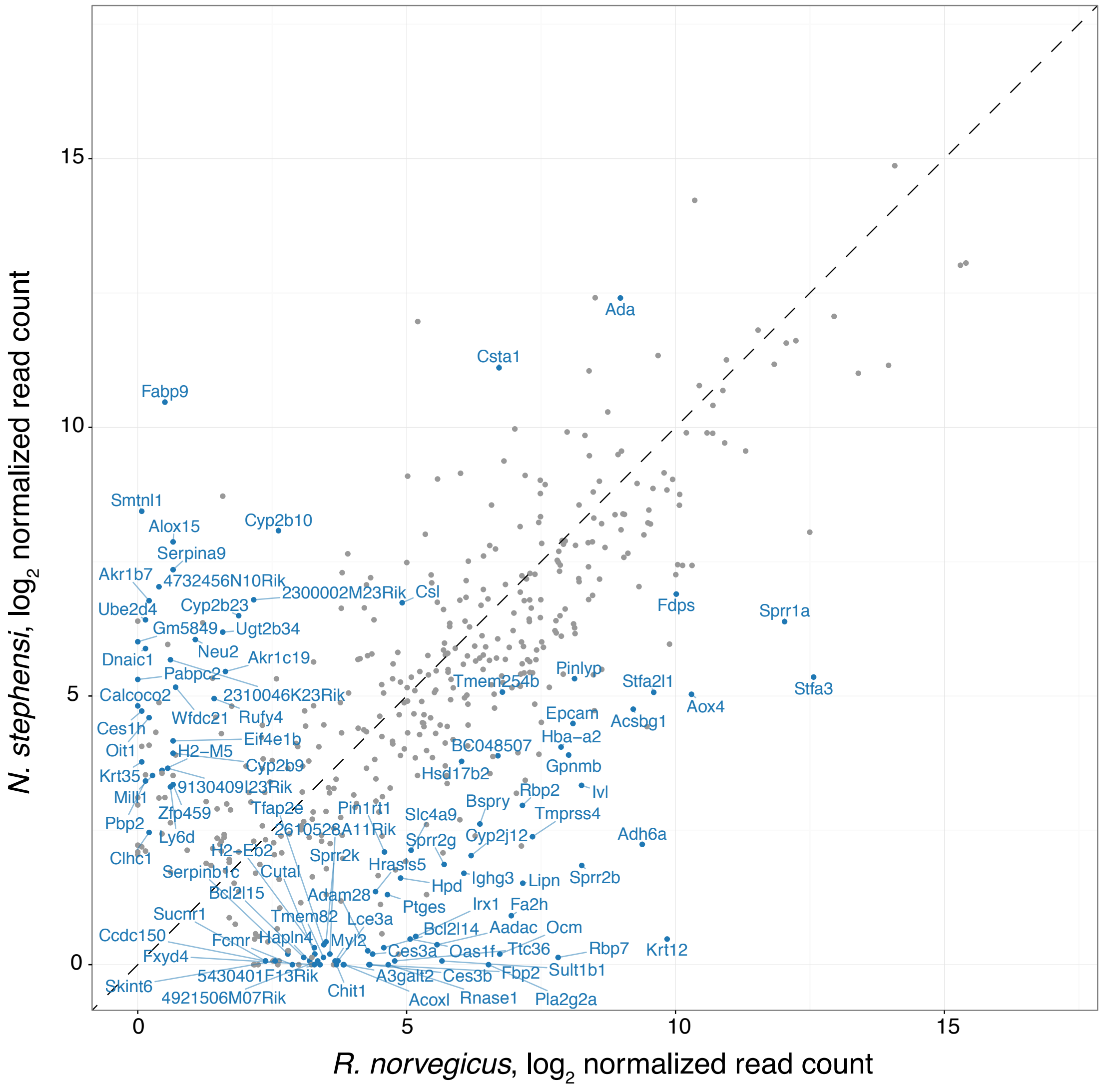

Figure S2. Differential abundance of 510 epidermal-specific transcripts between the two rodent species. Genes that had at least 100X higher average expression levels in rat foregut versus mouse jejunem are shown, with labels and color (blue) indicating differentially abundant (FDR < 0.002) genes.

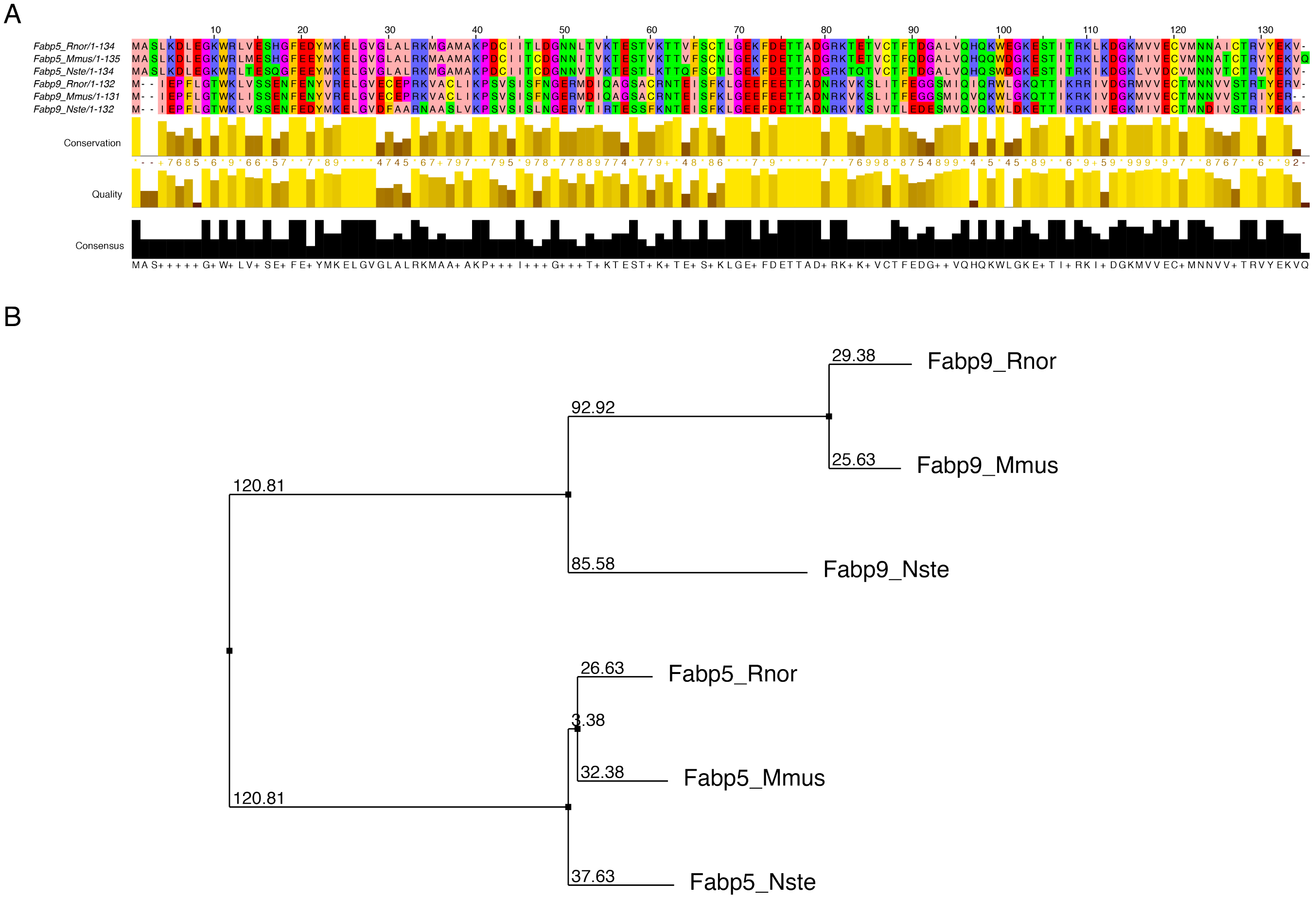

Figure S3. Multiple sequence alignment and phylogenetic tree of the abundant fatty acid binding proteins (Fabp5 and Fabp9) found in woodrat and laboratory rat foregut tissue. Protein sequences from Fabp5 and Fabp9 from *N. stephensi*, *R. norvegicus* and *M. musculus* were aligned with MAFFT Multiple Sequence Alignment Software Version 7 using the L-INS-I algorithm (mafft --reorder –auto input). A) Jalview alignment is shown with amino acid restudies colored according to their physicochemical properties and B) phylogenetic tree based on Neighbor Joining algorithm with a distance matix determined from BLOSUM 62 score.
